# The effect of multivitamin-multimineral supplementation on the health status of inbred Wistar and spontaneously hypertensive rat strains

**DOI:** 10.4102/jsava.v87i1.1324

**Published:** 2016-06-09

**Authors:** Rosemarie U. Höfler, Mahendra L. Channa, Anand Nadar

**Affiliations:** 1 School of Laboratory Medicine and Medical Sciences, University of KwaZulu-Natal, Westville Campus, South Africa

## Abstract

The nutraceutical industry has proliferated in recent years, with the most popular form of supplementation being the multivitamin-multimineral (MVMM) supplement. In the animal health sector, supplement use has also expanded. The objective of this study was to determine the effects of MVMM supplementation, beneficial or otherwise, on the general health status of the spontaneously hypertensive rat (SHR) strain, an animal model used in hypertension research. A commercially prepared MVMM supplement was given tri-weekly via oral dosing for 8 weeks to two groups of seven adult female SHR and Wistar rats. Their corresponding control groups were dosed with deionised water only. Systolic and diastolic blood pressure, fasting blood glucose, growth rate and food and water intake were measured weekly. At the end of 8 weeks, the animals were euthanased and a full blood profile, urine sodium to potassium ratio, blood urea nitrogen levels and total plasma cholesterol was measured for all groups. The results indicated that growth rate was higher for the SHR supplemented group. Supplementation also decreased diastolic blood pressure in both Wistar and SHR groups and increased red blood cell count and decreased total cholesterol in the SHR group. No adverse effects on the general health status of the animals were observed. MVMM supplementation may therefore be useful in aiding growth and delaying the onset of hypertension and its effects. It may also assist in the longevity of the breeding stock of SHR rats.

## Introduction

In recent years there has been a proliferation of the nutrient supplementation industry which, encouraged by various marketing claims, in 2007 was worth approximately 23.7 billion dollars in the USA alone (Marra & Boyar 2009). The most commonly purchased supplement was the multivitamin-multimineral (MVMM) supplement, showing a 3.9% increase in sales during that year alone (Marra & Boyar 2009). In the USA, multiple surveys known as the National Health and Nutrition Examination Survey (NHANES) were carried out over a period of years by the National Centre for Health Statistics to monitor dietary supplement use by its citizens. It revealed that between the years 1988–2006 there was a steady increase in MVMM supplement use in adults aged 20 and above, with women being the greatest consumers (Gahche *et al*. 2011). The primary role of pharmaceutically manufactured MVMM supplements is to target specific vitamin and/or mineral deficiencies that arise from primary or secondary malnutrition, which can be caused by a variety of disease conditions. Through high-impact marketing strategies otherwise healthy individuals are now also being encouraged to take these MVMM supplements in order to, as some claim, promote growth and general health, improve metabolism and enhance cognitive function (Ames, Atamna & Killilea 2005). It is also claimed that MVMM supplements act as a ‘nutritional insurance’ against the effects of ageing, fatigue, cancers and strokes, as well as chronic conditions such as hypertension, diabetes and cardiovascular disease (National Institutes of Health [NIH] 2015).

Predictably, the MVMM supplementation industry has now also spread into the veterinary and animal health sectors, with a large number of preparations and combinations being made available for livestock, poultry and companion animals, all with similar health-promoting purposes as for humans (National Animal Supplement Council [NASC] 2002). New MVMM formulations using nano-emulsion technology allow a multiple variety of supplements to be presented together in one suspension and delivered simultaneously in a single bolus (Srinivas *et al*. 2010). However, there is a concomitant need to include more research with specific focus on the effects and possible health risks associated with both human and animal MVMM supplementation (Comerford 2013). The NASC also raises the concern that there is no proper regulation for animal supplementation and many of the marketed supplements contain unsafe and/or unapproved ingredients, jeopardising the safety of the animals (NASC 2002).

The Biomedical Resources Unit (BRU) at the School of Laboratory Medicine and Medical Sciences, University of KwaZulu-Natal, *inter alia* breeds, houses and maintains a variety of laboratory rat strains like Wistar, Sprague Dawley, Dahl hypertensive and spontaneously hypertensive rats (SHR). The latter two strains serve as models for the study of salt-sensitive and genetic hypertension respectively, and are used extensively in research projects both at this and other research institutions. Unfortunately, as these animals age, they develop various sequelae of hypertension like lethargy, cardiovascular complications, decreased memory and learning as well as loss of libido and fertility, therefore requiring increased husbandry to maintain their health status (Azu 2013; Hernandez, Hoifodt & Terry 2003; Linz *et al*. 1999; Meneses & Hong 1998; Pepeu 2004).

The purpose of this current study was to determine whether administering a commercially available animal MVMM supplement to a cohort of Wistar and SHR strains would have any beneficial effect on their overall general health status. The associated objective was to assist the BRU in deciding whether supplementation is indeed necessary for maintaining optimal health of the breeding stock.

## Materials and methods

Twenty-eight female rats supplied by the BRU, 14 SHR and 14 Wistar (WIS), weighing approximately 150 g each, were used for the study. The SHR were randomly divided into supplemented (SHR S) and unsupplemented (SHR) groups of seven animals each. The Wistar animals were also similarly divided into supplemented (WIS S) and unsupplemented (WIS) groups of seven animals each. All animals were maintained at a temperature of 22 °C ± 1 °C, exposed to a 12-hour light-dark cycle, and each group was accommodated in a 50 cm × 40 cm × 18 cm polycarbonate cage. The study was designed around a 7-day time line repeated for the duration of 8 weeks. All animals had *ad libitum* access to water and commercial rat chow (Meadow Feeds™ standard maintenance rat chow). General appearance and behaviour were monitored daily. Body mass was monitored weekly and food and water intake and urine output were monitored weekly by placing the animals individually in metabolic cages. Blood pressure and fasting blood glucose were measured weekly for all rats.

### Supplement selection and dosage calculation

The Byboost™ MVMM supplement with added copper (see Table 1 for composition) was chosen, supplied by Bayer Health Care (Pty) Ltd, South Africa.

**TABLE 1:** Composition of Byboost™ multivitamin-multimineral supplement with added copper (per litre).

Active ingredient	Quantity
Vitamin A	1 000 000 IU
Vitamin D_3_	152 000 IU
Vitamin E (as α-tocopherol acetate)	15 000 mg
Vitamin B_1_	2700 mg
Vitamin B_2_	1000 mg
Vitamin B_3_	3500 mg
Vitamin B_5_ (Calcium pantothenate)	1250 mg
Vitamin B_6_	1250 mg
Folic acid	1250 mg
Vitamin B_12_	13 000 µg
Vitamin C	1000 mg
Vitamin H (Biotin)	3750 µg
Vitamin K (Menadione sodium bisulfite)	410 mg
Iodine	1000 mg
Selenium	300 mg
Cobalt	1350 mg
Copper (Metal amino acid chelate)	3400 mg
Manganese (Metal amino acid chelate)	2000 mg
Zinc (Metal amino acid chelate)	6000 mg
Lysine hydrochloride	2200 mg
Methionine	3500 mg
Amino acids	2100 mg
Essential fatty acids	1500 mg

*Source*: Bayer South Africa, n.d., *Byboost Sheep and Goats + Copper*, package insert, Bayer, Isando

The Byboost supplement was chosen on the basis of its formulation; it made use of nanotechnology, a fairly recent application in the field of nutritional sciences, which allows for a single bolus containing a variety of supplements to be administered (Srinivas *et al*. 2010). Extensive research is still being conducted to determine the efficacy of these nanoparticles, particularly nanominerals, in the livestock industry, where they are used to increase bioavailability to enhance reproduction, growth and immunity (Rajendran 2013). Research conducted thus far shows promising results; intracellular pathways and cellular components can now be reached much more easily and effectively, which may lead to and provide new preventative and therapeutic methods (Srinivas *et al*. 2010).

The dosage of the supplement was calculated according to the body weight of the rats. Briefly, 0.5 mL of the supplement was diluted in deionised water to make up 5 mL. Each rat was then orally dosed with 0.5 mL of this composition. The calculated dosage fell within the range of the recommended daily allowance for the rats according to the recommendations for nutrient requirements of laboratory animals (Subcommittee on Laboratory Animal Nutrition 1995) for rats and mice.

### Growth rate

Each rat was individually weighed each week for 8 weeks and the increase in body mass was expressed as a percentage increase based on the weight of the rat from day zero of the study.

### Systolic and diastolic blood pressure measurements

Systolic (SBP) and diastolic (DBP) blood pressure was monitored weekly for each group using the non-invasive tail cuff method (IITC Instruments, USA). This is a validated and standardised method in laboratories to measure rodent hypertension (Somova, Channa & Khan 1999).

### Food and water intake

Food intake, water intake and urine excretion volume for all groups were measured weekly over a 24-hour period by placing rats individually in metabolic cages (Techniplast™, Italy).

### Fasting blood glucose levels

Each week animals were fasted overnight and blood glucose levels were measured using the Accu-Check Active^®^ Blood Glucose Monitor (Roche, Germany) with accompanying test strips.

### Blood sample collection

After week eight, animals were anaesthetised by a halothane inhalation and exsanguinated by cardiac puncture. Approximately 7 mL of blood was collected from each animal by this method. The parameters below were measured once after the 8-week protocol.

### Total cholesterol and haematological status

A full blood count was conducted immediately using 1 ml of blood (Beckman Coulter, USA). Total cholesterol was measured immediately after sacrificing using the Accutrend Plus^®^ meter (Cobas, USA).

### Urinary sodium to potassium ratio and blood urea nitrogen

The urinary sodium, potassium and creatinine parameters were measured at week eight using the CX 3 Electrolyte Analyser (Beckman Coulter, USA). Blood urea nitrogen (BUN) was analysed once in plasma samples collected after the end of the 8-week protocol (Global Clinical & Viral Labs).

### Statistical analysis

Statistical analysis was performed using GraphPad Instat Version 5.00 and StatView Version 5.0 software. Repeat measures analysis of variance (ANOVA) was used to analyse blood pressure and growth rate and independent sample t-tests were used for the remaining data. All values were expressed as a mean ± SEM (standard error of the mean), where *p* < 0.05 was considered significant. The data were plotted using GraphPad (V 5.00) software.

## Ethical considerations

This study was approved by the Biomedical Research Ethics Administration of the University of KwaZulu-Natal, Westville Campus, Durban, South Africa. Principles of laboratory animal care (NIH 2010) were followed. Ethics No. 098/13/Animal.

## Results

### Animal growth

**FIGURE 1:**
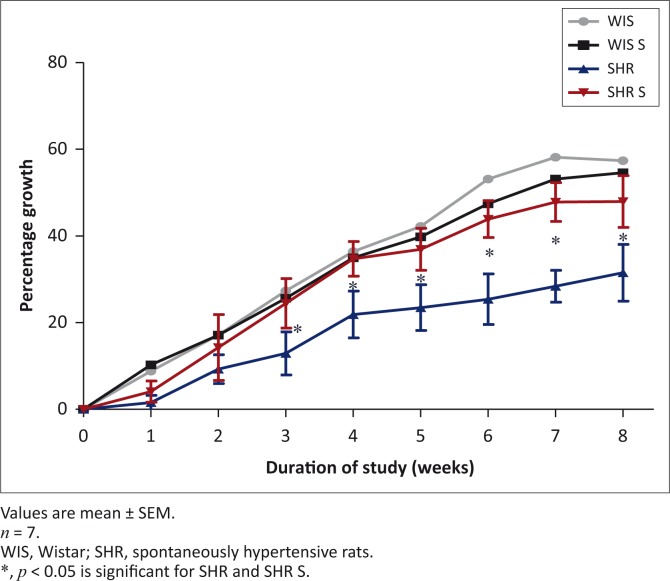
The effect of multivitamin-multimineral supplementation on growth rate in spontaneously hypertensive and Wistar rats.

The growth rate for all the WIS and SHR groups was similar at the start of the study. However, there was a significant increase in growth rate from the third week until the eighth week in the SHR S group when compared with the SHR. At the end of week eight, the SHR S group had a growth rate 16.4% higher than the non-supplemented group. MVMM supplementation did not make any significant difference in the growth rate of the WIS strain, which normally exhibits a higher growth rate than the SHR strain (Figure 1).

### Systolic blood pressure

**FIGURE 2:**
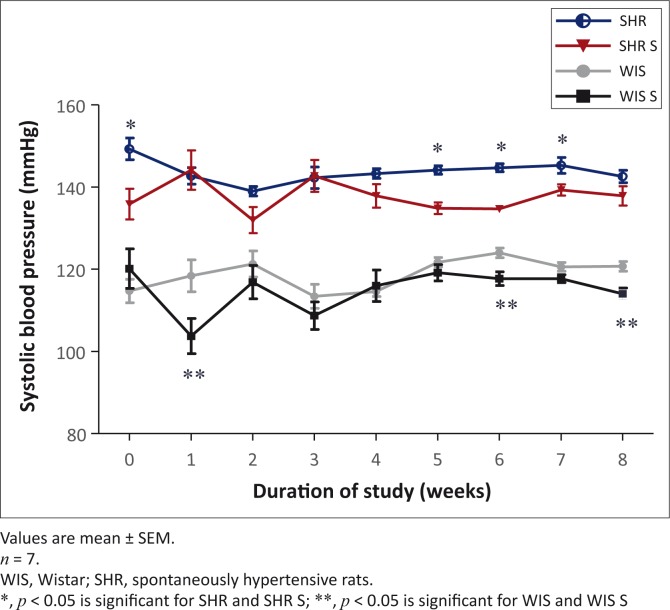
The effect of multivitamin-multimineral supplementation on systolic blood pressure in spontaneously hypertensive and Wistar rats.

In keeping with the primary characteristic feature of the SHR model, the SBP was in the hypertensive range throughout the study, in contrast to the normotensive WIS strain. The SHR can be classed as Stage 1 hypertensive whilst the WIS rats were normotensive (World Health Organization [WHO] 2013). Supplementation had no major effect on the SBP in both groups except during week one in the WIS S group and week six in both the WIS S and SHR S groups, both showing a transient decrease (Figure 2).

### Diastolic blood pressure

**FIGURE 3:**
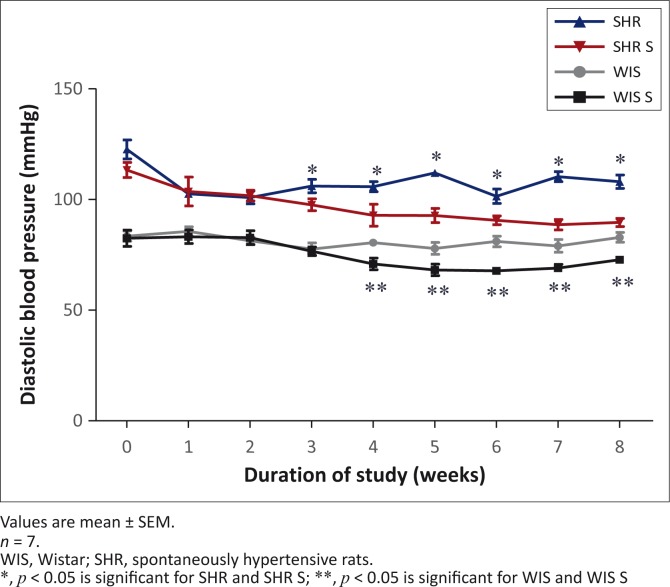
The effect of multivitamin-multimineral supplementation on diastolic blood pressure in spontaneously hypertensive and Wistar rats.

As with the SBP, the DBP was also in the hypertensive range in the SHR throughout the study, in contrast to the normotensive WIS strain. The SHR can be classed as Stage 1 hypertensive whilst the WIS rats were normotensive (WHO 2013). At week eight, there was an average SBP difference of 25 mmHg between the strains. Supplementation had a significant DBP-lowering effect in both the SHR S and WIS S groups in weeks four, five, seven and eight. At week eight, there was a difference of 7 mmHg between the WIS S and WIS groups and a difference of 16 mmHg between the SHR S and SHR groups (Figure 3).

### Haematological parameters

No significant differences in white blood cell (WBC) count and haematocrit (HCT) were observed amongst all four groups. Haemoglobin (Hb) showed a significant strain difference between the SHR and WIS groups with supplementation having no significant effects in either strain. The SHR S group showed a significantly higher red blood cell (RBC) count compared to the SHR group, an effect that was not observed in either of the WIS groups (Table 2).

**TABLE 2:** Effect of multivitamin-multimineral supplementation on red blood cell count, white blood cell count, haematocrit and haemoglobin in spontaneously hypertensive and Wistar rats for week eight.

Groups	RBC (106/μL)	WBC (103/μL)	HCT (%)	Hb (g/L)
WIS	7.48 ± 0.28	4.2 ± 0.5	41.5 ± 1.10	148 ± 3.89*
WIS S	7.67 ± 0.14	3.6 ± 0.3	42.2 ± 1.07	152 ± 3.52
SHR	7.79 ± 0.12†	4.2 ± 0.3	39.2 ± 0.68	138 ± 2.43*
SHR S	8.26 ± 0.16†	3.8 ± 0.4	41.1 ± 0.86	145 ± 3.12

Values are mean ± SEM. *n* = 7. WIS, Wistar; SHR, spontaneously hypertensive rats; RBC, red blood cell; WBC, white blood cell; HCT, haematocrit; Hb, haemoglobin.

* *p* < 0.05 is significant for WIS and SHR;

† *p* < 0.05 is significant for SHR and SHR S

### Fasting blood glucose and total cholesterol

No significant difference in fasting blood glucose levels was observed in any of the four groups at week eight. There was a significant strain difference, as the SHR had higher total cholesterol than the WIS. Supplementation seems to have slightly but significantly lowered total cholesterol in the SHR S group (Table 3).

**TABLE 3:** Effect of multivitamin-multimineral supplementation on total cholesterol and fasting blood glucose in spontaneously hypertensive and Wistar rats for week eight.

Groups	Total cholesterol (mmol/L)	Fasting glucose (mmol/L)
WIS	4.29 ± 0.06*	5.1 ± 0.11
WIS S	4.55 ± 0.26	4.9 ± 0.18
SHR	4.51 ± 0.03*†	4.4 ± 0.23
SHR S	4.30 ± 0.04†	4.9 ± 0.19

Values are mean ± SEM. *n* = 4 for total cholesterol; *n* = 7 for fasting glucose. WIS, Wistar; SHR, spontaneously hypertensive rats.

* *p* < 0.05 is significant for WIS and SHR

† *p* < 0.05 is significant for SHR and SHR S

### Food and water intake and urine collection

There was no significant difference observed in the food intake and urine output in all four groups by the end of week eight. Water intake was significantly higher in the SHR strain than in the WIS, with supplementation having no significant effect on water intake in either group (Table 4).

**TABLE 4:** Effect of multivitamin-multimineral supplementation on food, water intake and urinary output in spontaneously hypertensive and Wistar rats for week eight.

Groups	Food intake (g)	Water intake (mL)	Urinary output (mL)
WIS	17.1 ± 1.73	19.4 ± 2.02*	10.3 ± 0.95
WIS S	17.1 ± 1.73	24.4 ± 0.71	10.5 ± 0.81
SHR	16.6 ± 0.87	29.4 ± 2.10*	10.3 ± 0.92
SHR S	16.9 ± 0.83	30.0 ± 4.08	13.4 ± 2.53

Values are mean ± SEM. *n* = 7. WIS, Wistar; SHR, spontaneously hypertensive rats.

* *p* < 0.05 is significant for WIS and SHR

### Urinary sodium to potassium ratio and blood urea nitrogen

There was no significant difference observed in urinary creatinine in any of the four groups at the end of week eight. BUN and sodium to potassium ratio showed a significant strain difference, as the SHR had higher sodium to potassium ratio and BUN levels than the WIS. Supplementation had no effect on either of these parameters in either group (Table 5).

**TABLE 5:** Effect of multivitamin-multimineral supplementation on urinary creatinine, sodium to potassium ratio and blood urea nitrogen in spontaneously hypertensive and Wistar rats for week eight.

Groups	Creatinine (mmol/L)	Na/K ratio (mmol/L)	BUN (mmol/L)
WIS	3.82 ± 0.95	0.52*	6.5 ± 0.51*
WIS S	5.34 ± 0.79	0.51	7.6 ± 0.51
SHR	2.24 ± 0.43	0.75*	8.3 ± 0.30*
SHR S	3.35 ± 0.71	0.80	8.2 ± 0.44

Values are mean ± SEM. *n* = 7 for creatinine and Na^+^/K^+^ ratio; *n* = 6 for BUN. WIS, Wistar; SHR, spontaneously hypertensive rats; BUN, blood urea nitrogen.

* *p* < 0.05 is significant for Wistar and spontaneously hypertensive rats

## Discussion

The MVMM supplementation increased growth by 16.4% in the SHR S group. Individual animal food intake, monitored in a metabolic cage once a week, showed no difference in the SHR groups. However, variations in food intake influenced by MVMM supplementation may have possibly occurred during communal feeding. This together with other anabolic responses may account for this growth difference. Vitamins D and B_6_ have been found in previous rat studies to increase growth and growth hormone release (Huber & Gershoff 1965; Steenbox & Herting 1955). Both of these vitamins are present in the Byboost™ supplement and may be the cause of the higher growth rate. At this stage it cannot be explained why the supplement had no effect on the growth rate of the WIS strain.

The significant DBP-lowering effect of MVMM supplementation was observed after four weeks in both the WIS S and SHR S groups (Figure 3). This change actually brought the DBP in the SHR S group from Stage 1 hypertensive to almost borderline hypertensive levels (WHO 2013). The lowering of the DBP in particular indicates that supplementation may have more of an effect on the vasculature and peripheral resistance rather than on the cardiac output, as heart rates were not altered significantly in any of the four groups. Supplementation had a greater DBP-lowering effect in the SHR than in the WIS. The lowering of the DBP by between 7 mmHg and 16 mmHg is equivalent to the effects of some of the antihypertensive drugs currently available (Limas & Freis 1972; Thomopoulos, Parati & Zanchetti 2015). Previous studies showed the individual antihypertensive effects of vitamins B_6_, E and C in SHRs, mainly on the SBP through effects on the renal vasculature (Vasdev *et al*. 1991, 2001, 2002). All three of these vitamins are found in the Byboost™ supplement given to the supplemented groups. There are also numerous minerals in the Byboost™ supplement that have the ability to alter blood pressure. Selenium and copper are known to strengthen cellular antioxidant enzymes like GPX, catalase and superoxide dismutase levels, as well as the nitric oxide vasodilatory mechanism that plays a major role in blood pressure regulation (Arthur, McKenzie & Beckett 2003; Maggini *et al*. 2007; Ognjanovic *et al*. 2008). Zinc, copper, manganese and other minerals may have direct blood pressure lowering effects (Klahr 2001; Loyke 1997, 2002; Rodrigo *et al*. 2007). The overall effect of the supplementation was to lower DBP and to perhaps delay the onset of severe hypertension. Continued supplementation may also have the potential to prevent end organ damage in hypertension.

The haematological parameters, except the RBC count and haemoglobin, showed no significant differences between the WIS and SHR strains nor were they altered by supplementation. There was a significant strain difference in haemoglobin levels, as the SHRs had a significantly lower level than the WIS group. Supplementation resulted in a slight but significant increase in the RBC count in the SHR group but had no effect on the haemoglobin levels. The Byboost™ supplement contains essential fatty acids; a previous study has shown that an increased intake of fatty acids, specifically n-3 poly-unsaturated fatty acids, results in an increase in RBC in SHRs (Bacova *et al*. 2013).

Fasting blood glucose and total cholesterol are parameters indicative of carbohydrate and lipid metabolism. There was a significant strain difference between the WIS and SHR groups in total cholesterol levels. In the SHR S, it appeared that the supplement had a slight but significant cholesterol-lowering effect. Supplementation had no effect on the fasting blood glucose levels.

The sodium to potassium ratio, plasma creatinine, BUN and water intake are parameters typically indicative of kidney function (Duarte, Zhang & Ellis 2001; Gowda *et al*. 2010). Creatinine levels showed no significant differences between all groups whereas the sodium to potassium ratio, water intake and BUN were significantly higher in the SHR strain. This indicates higher sodium retention, expansion of plasma volume and a decreased glomerular function in the SHR. It also may in part indicate the renal contribution to the pathogenesis of hypertension in the SHR group (Feld *et al*. 1990; Karam *et al*. 1996; Purkerson, Hoffsten & Klahr 1976). Supplementation had no significant effect on the renal parameters. MVMM supplementation with its DBP-lowering effects therefore appears to have had greater effect on the cardiovascular system and/or the central nervous system rather than on the renal system.

## Conclusion

In conclusion, the MVMM supplementation had significant metabolic and cardiovascular effects in the SHR group. It increased growth and caused a significant reduction in the DBP of both the SHR S and WIS S groups over the 8-week period. It also to a lesser extent lowered total cholesterol and increased RBC count in the SHR S group. Although renal function in the SHR group was altered, supplementation had no positive or negative effects. No deleterious effects on the health status of the animals as a result of the supplementation were observed.

The pathogenesis of hypertension is both polygenic and multifactorial in the SHR model. It may be accompanied by various metabolic aberrations, including alterations in trace element and vitamin metabolism, which may in turn contribute towards the pathogenesis of hypertension or, alternatively, may arise from it. A marginal MVMM deficiency therefore cannot be excluded in the SHR strain; this could possibly account for its positive response to the supplementation.

New-age MVMM supplementation, using nanotechnology, incorporated in the diets, especially in the growth phase of young animals, may be effective in maintaining general growth and health status. It may be also important in delaying the onset of the effects of hypertension in the breeding stock of SHR rats.
